# Chitosan oligosaccharides in combination with *Agaricus blazei* Murill extract reduces hepatoma formation in mice with severe combined immunodeficiency

**DOI:** 10.3892/mmr.2015.3454

**Published:** 2015-03-09

**Authors:** MING YANG YEH, HUNG SHENG SHANG, HSU FENG LU, JASON CHOU, CHUN YEH, JIN BIOU CHANG, HSIAO FANG HUNG, WAN LIN KUO, LUNG YUAN WU, JING GUNG CHUNG

**Affiliations:** 1 Department of Medical Education and Research, Cheng Hsin General Hospital, Taipei 112, Taiwan, R.O.C.; 2 Department of Pathology, National Defense Medical Center, Division of Clinical Pathology, Tri-Service General Hospital, Taipei 112, Taiwan, R.O.C.; 3 Departments of Clinical Pathology, Cheng Hsin General Hospital, Taipei 112, Taiwan, R.O.C.; 4 Departments of Anatomical Pathology, Cheng Hsin General Hospital, Taipei 112, Taiwan, R.O.C.; 5 Division of Gastroenterology, Cheng Hsin General Hospital, Taipei 112, Taiwan, R.O.C.; 6 Department of Medical Laboratory Science and Biotechnology, Yuanpei University, Hsinchu 300, Taiwan, R.O.C.; 7 Department of Medical Technology, Jen-Teh Junior College of Medicine, Nursing and Management, Miaoli 356, Taiwan, R.O.C.; 8 Department of Biology, Ching Cheng High School, Changhua 500, Taiwan, R.O.C.; 9 School of Chinese Medicine for Post Baccalaureate, I Shou University, Kaohsiung 840, Taiwan, R.O.C.; 10 Department of Biological Science and Technology, China Medical University, Taichung 404, Taiwan, R.O.C.; 11 Department of Biotechnology, Asia University, Taichung 404, Taiwan, R.O.C.

**Keywords:** chitosan, *Agaricus blazei* Murill, vascular endothelial growth factor

## Abstract

Chitosan and *Agaricus blazei* Murill (ABM) extracts possess antitumor activities. The aim of the present study was to investigate whether chitosan, ABM extract or the two in combination were effective against tumors in tumor-bearing mice. The mice were subcutaneously injected with SK-Hep 1 cells and were then were divided into the following six groups: Group 1, control group; group 2, chitosan 5 mg/kg/day; group 3, chitosan 20 mg/kg/day; group 4, ABM (246 mg/kg/day) and chitosan (5 mg/kg/day) combined; group 5, ABM (984 mg/kg/day) and chitosan (20 mg/kg/day) combined; and group 6, ABM (984 mg/kg/day). The mice were treated with the different concentrations of chitosan, ABM or combinations of the two for 6 weeks. The levels of glutamic oxaloacetic transaminase (GOT), glutamic pyruvic transaminase (GPT) and vascular endothelial growth factor (VEGF), and tissue histopathological features were examined in the surviving animals. Based on the results of the investigation, the treatments performed in groups 2, 3 and 4 were identified as being capable of reducing the weights of the tumors, however, group 4, which was treated with chitosan (5 mg/kg/day) in combination with ABM (246 mg/kg/day) was able to reduce the levels of GOT and VEGF. As a result, treatment with chitosan in combination with ABM may offer potential in cancer therapy and requires further investigation.

## Introduction

Chitosan is a mucopolysaccharide, which is closely associated with cellulose and is obtained by the deacetylation of chitin, which is the predominant compound present in the exoskeleton of crustaceans (1). The biopolymer chitosan is defined as containing <50% N-acetyl-glucosamine, whereas if the number of N-acetylglucosamine units is >50%, the biopolymer is termed chitin (2). The biological activity of chitosan is dependent on its molecular weight, extent of deacetylation, chitosan derivatization, proportion of glucosamine units, pH and its target (3–5).

Chitosan has been previously demonstrated to exhibit therapeutic effects in the inhibition of inflammation in asthma (6–9), in the strengthening of bones in osteoporosis (10,11), as an antibacterial agent (7), a vector for gene delivery (12,13), an antifungal agent (14), an anti-malaria agent (15) and a homeostatic agent in wound dressings (16). Low molecular weight (LMW) and water-soluble chitosan, are efficient colloidal drug carriers (17) due to high levels of water solubility, non-toxicity, biocompatibility, biodegradability, and bioadhesive and absorption enhancing properties (18). In addition, the potential biological activities of LMW chitosan, including its antioxidative and antitumorigenic properties, make it a suitable candidate for biomedical applications (14,18,19). Previous studies have indicated that chitosan exhibits antitumorigenic activity (18,20). Mushrooms are another natural product with medicinal uses, and have been used for several years in Asian countries and their use is increasing in western countries. The number of mushroom species on the planet has been estimated at ~ 140,000, however, suggesting that only 10% of species have been identified (21). Under the assumption that only 5% of the unknown species of mushrooms will be beneficial to humans, this indicates that ~7,000 useful species remain to be identified (22). Among the known species, the proportion which have been thoroughly investigated remains low. Mushrooms require antibacterial and antifungal compounds in order to survive in their natural environment (23), and these can be isolated from the mushrooms to provide potential therapeutic benefits for humans (24). Previous studies in Asia and eastern Europe have indicated that mushrooms may be important in preventing and treating cancer (25), and the antitumor effects of several mushroom extracts and isolated compounds have been demonstrated in tumor cell systems and in animal assays (26–33).

Mushroom extracts have been identified as immunological, hypocholesterlemic, antiviral, antibacterial, anticarcinogenic and anti-inflammatory (34). *Agaricus blazei* Murill (ABM) is an edible mushroom, which is native to Brazil and is cultivated in several countries, including Taiwan, Japan, Korea, China and Indonesia (25). ABM has been reported to possess antitumor activity (35,36), however, the function of ABM in SK-Hep 1 hepatoma cells in mice with severe combined immunodeficiency (SCID) has not been investigated.

The aim of the present study was to investigate whether ABM extract or LMW chitosan were effective antitumorigenic compounds, and to determine whether the combination of ABM and chitosan was more effective than either of the compounds alone in reducing the size of tumors in mice injected with hepatoma cells.

## Materials and methods

### Animals and housing conditions

Animals were maintained in accordance with the guidelines approved by the National Science Council of the Republic of China and the Committee for the Purpose of Control and Supervision of Experiments on Animals. Experiments are performed in accordance with the law, regulations and guidelines for animal experiments in Taiwan, which are in agreement with the Declaration of Helsinki. The investigations involving mice were approved by the Institutional Animal Care and Use Committee of Chen Hsin General Hospital (Taipei, Taiwan; CHIACUC 102-18). A total of 60 SCID mice (male, weighing 22–26 g, four-weeks-old) were obtained from BioLASCO Taiwan Co., Ltd. (Taipei, Taiwan). The mice were earmarked and housed in polypropylene cages (five animals/cage) covered with metallic grids in a room maintained under constant environmental conditions, with air filter tops in a filtered laminar air flow, an ambient temperature of 22±3°C, relative humidity of 55±15% and with a 12-h light-dark cycle for a 2-week acclimatization period. The mice received autoclaved water and laboratory pellet chow *ad libitum* (37).

### Chitosan and ABM preparation

Chitosan powder (molecular weight, 50,000–190,000; cat. no. 448869; Sigma-Aldrich, St. Louis, MO, USA) was suspended in 0.2 ml distilled water at 50°C for 10 min, and then cooled to room temperature and stirred for 1 h at 200 rpm using a TS-560 orbital shaker (Yihder Technology Co., Ltd., Taipei, Taiwan) (low dose 5 mg/kg/day; high dose 20 mg/kg/day). ABM powder was obtained from S. Canaan Biotechnology Development Co. (Taipei, Taiwan) and was separately suspended in 6 ml distilled water at 60°C for 10 min, then cooled to room temperature and stirred for 5 h at 200 rpm to form solutions of 246 mg/kg body weight/0.2 ml or 984 mg/kg body weight/0.2 ml which were 10 or 40 times of therapy doses cited by package insert. The ABM supernatant solution was then filtered, freeze dried, and stored at −50°C until use, as previously described (38).

### Hepatoma formation using SK-Hep 1 cells in SCID mice and treatment with ABM and chitosan

All of the mice were injected subcutaneously with SK-Hep 1 cells (Food Industry Research and Development Institute, Hsunchu, Taiwan) (3×10^7^ cells/mouse) in the dorsal area. Tumors were allowed to develop for 2–3 weeks (week 0), and mice with tumors measuring 1–3 mm in diameter were divided into the following six groups (10 mice/group): Group 1, control group (distilled water); group 2, chitosan 5 mg/kg/day; group 3, chitosan 20 mg/kg/day; group 4, ABM (246 mg/kg/day) and chitosan (5 mg/kg/day) combined; group 5, ABM (984 mg/kg/day) and chitosan (20 mg/kg/day) combined; and group 6, ABM (984 mg/kg/day). Following 6 weeks of treatment, the levels of serum glutamic oxaloacetic transaminase (GOT), glutamic pyruvic transaminase (GPT) and vascular endothelial growth factor (VEGF) were examined in the surviving animals. The mice were then sacrificed using CO_2_, and the tumors were surgically excised and weighed, prior to histopathological analysis.

### Serum biomarkers

Whole blood (0.5–1 ml) was collected from each mouse via heart puncture. The collected blood was centri fuged (2,000 × g) for 10 min using the Kubota 2420 centrifuge (Kubota, Fujioka, Japan). The serum levels of GOT and GPT were analyzed using a D×C 800 clinical chemistry analyzer with kits (GOT catalog no., M307050; GPT catalog no., M312240) purchased from Beckman Coulter (Brea, CA, USA).

The quantification of murine VEGF in the serum was determined using a mini ELISA development kit (900-M99; cat. no. 0812099-M), according to the manufacturer’s instructions (PeproTech, Inc., Rocky Hill, NJ, USA). Briefly, for ELISA, undiluted standard (1.5 ng/ml; from the ELISA kit) served as the highest standard level and the calibrator diluents served as the zero standard. For the measurement of VEGF, 100 *μ*l undiluted sample or standard was added to each well of the ELISA plate and incubated at room temperature for a minimum of 2 h. Aspiration and washing of the plate with buffer (0.05% Tween-20 in PBS; PeproTech) were performed four times. The detection antibody (purified rabbit anti-VEGF; 0812099-M; PeproTech, Inc.) was diluted in diluents (0.05% Tween-20 + 0.1% bovine serum albumin in phosphate-buffered saline; PeproTech) to a concentration of 0.5 *μ*g/ml. A total of 100 *μ*l/well was added and the plate was incubated at room temperature for 2 h. Aspiration and washing of the plate were performed four times, and 5.5 *μ*l avidin-horseradish peroxidase conjugate (1:2,000) was added to the diluent to a total volume of 11 ml, of which 100 *μ*l was added per well and incubated for 30 min at room temperature. The plate was then aspirated and washed four more times, and 100 *μ*l substrate solution was added to each well prior to incubation at room temperature for color development. The color development was assessed using an ELISA plate reader (Bio Rad Laboratories, Inc., Hercules, CA, USA) at 405 nm with the wavelength correction set at 650 nm.

### Histopathology

At the end of the 6 weeks treatment, the survival rates of the mice were assessed. Histological analysis of the liver tissues were also performed as follows: The tissue samples were rinsed with 0.9% saline solution (Jye-Jiunn, Taipei, Taiwan) and fixed in 10% formalin (Avantor Performance Materials, Deventer, Netherlands). The liver sections were then prepared and processed (TP1020; Leica Microsystems KK, Tokyo, Japan) as follows: The sections were incubated twice with 10% neutral buffered formalin for 30 min each, 75% alcohol (Jye-Jiunn) at room temperature for 1 h, 85% alcohol at room temperature for 1 h, twice with 95% alcohol at room temperature for 1 h, twice with 100% alcohol at 40°C for 1 h, twice with xylene (Surgipath, Leica Microsystems, Inc., Buffalo Grove, IL, USA) at 40°C for 1 h and in molten wax (Surgipath, Leica Microsystems, Inc.) at 60°C for 30 min repeated 4 times. The samples were embedded in paraffin (Leica Biosystems Richmond, Inc., Richmond, IL, USA), sectioned (4 *μ*m), placed on frosted glass slides (Muto Pure Chemicals Co., Ltd., Tokyo, Japan), dried using a 70°C hot plate (Yihder Technology Co., Ltd.) for 30 min and stained with hematoxylin and eosin (H&E; Muto Pure Chemicals Co., Ltd.).

### Statistics

The data are presented as the mean ± standard deviation. One way analysis of variance was used to determine significant differences between the control and treated groups. Student’s t-test was used to compare the means from two independent groups. P<0.05 was considered to indicate a statistically significant difference.

## Results

Following treatment for 6 weeks, the survival rates of the mice in the different groups were as follows: Group 1, 90% (9/10); group 2, 100% (10/10); group 3 100% (10/10); group 4, 100% (10/10); group 5, 70% (7/10); and group 6, 70% (7/10). A blood sample was not obtained from this animal, however, the tumor was weighed.

The serum concentrations of the GOT and GPT biochemical markers were analyzed to evaluate liver function. The levels of VEGF, which is a key angiogenic factor, were also examined. In addition, tumor weights and histopathological changes were evaluated.

**Figure 1:**
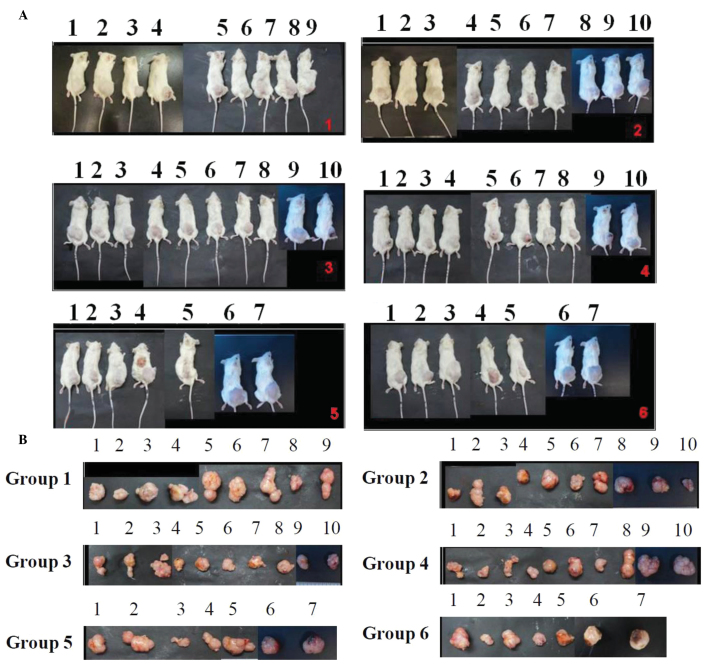
Treatment with chitosan and ABM affects subcutaneously implantated SK-Hep 1 cells in SCID mice *in vivo*. SK-Hep 1 cancer cells were inoculated subcutaneously into the dorsal area of each mouse. At 2 3 weeks after inoculation, each mouse had produced one palpable tumor of 1–3 mm in diameter. The mice were randomly divided into six groups, each containing 10 animals, one of which animal did not survive to the end of the experiment. Images of the (A) live mice and (B) representative tumors were captured. Group 1, control group; group 2, chitosan 5 mg/kg/day; group 3, chitosan 20 mg/kg/day; group 4, ABM (246 mg/kg/day) and chitosan (5 mg/kg/day) ; group 5, ABM (984 mg/kg/day) and chitosan (20 mg/kg/day); group 6, ABM (984 mg/kg/day). ABM, *Agaricus blazei* Murill; SCID, severe combined immunodeficiency.

Following the injections with SK-Hep 1 cells to induce tumor growth, the mice were orally administered different doses of LMW chitosan combined with ABM. Following 6 weeks treatment, blood samples were collected from all the surviving mice, which were then sacrificed, and the weights of the tumors were assessed (Fig. 1). The tumor weights were 4.81±1.84, 2.83±1.23, 2.56±1.65, 2.15±1.33, 4.59±2.11 and 3.79±2.39 g for groups 1-5, respectively. Groups 2, 3 and 4 exhibited significantly reduced tumor growth compared with the control group (P<0.05; Table I). No significant differences were observed among these three groups in the reduction of tumor weights.

The concentration of GOT was significantly reduced in group 2 (91±19 IU/l; P=0.020) and group 3 (99±26 IU/l; P=0.033) compared with the control group (162±80 IU/l) following 6 weeks treatment. This suggested that treatment with 20 mg chitosan or 5 mg chitosan combined with 246 mg ABM improved liver function. The levels of GOT in the control and ABM-only (139±86 IU/l) treatment groups were not significantly different. The mice treated with increasing doses of chitosan or ABM did not exhibit any gradual elevation or reduction in serum levels of GPT. The GPT concentrations were increased (35±31 IU/l) following administration of 5 mg chitosan treatment compared with that in the control group (17±4 IU/L), however, this was not statistical significant (P=0.054; Table I). The concentration of VEGF was significantly different in group 3 (0.459±0.096 ng/ml; P=0.0191) and group 5 (0.439±0.039 ng/ml, P=0.0167) compared with the control group (0.572±0.054 ng/ml) after 6 weeks (Table I).

**Figure 2:**
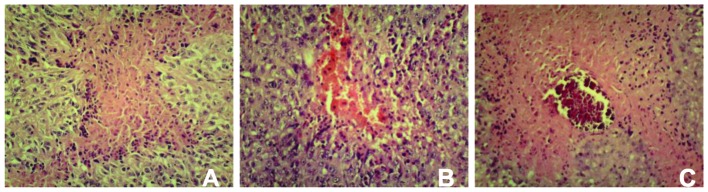
Histopathological images from group 2 (5 mg chitosan). (A) Irregular shapes of necrotic areas were composed of central eosinophilic cell debris and peripheral viable tissues. (B) Hemorrhages were observed in the center of certain necrotic areas. (C) Certain necrotic areas exhibited scarlet calcification. (hematoxylin and eosin staining; magnification, ×200).

The histopathological assessments were performed in the control and experimental groups. The tumor sections were stained with H&E and exhibited dark eosinophilic cytoplasms and small or large, darkly stained nuclei. The tissues from the mice in the control and experimental groups exhibited necrosis, calcification and hemorrhaging (Fig. 2). Irregular shapes of focal necrotic areas and loss of normal architecture were characterized by necrotic cells with eosinophilic cell debris and peripheral viable tissues. Foci of hemorrhage and scarlet calcification were frequently observed in the center of certain necrotic areas (Table II).

## Discussion

**Figure 3:**
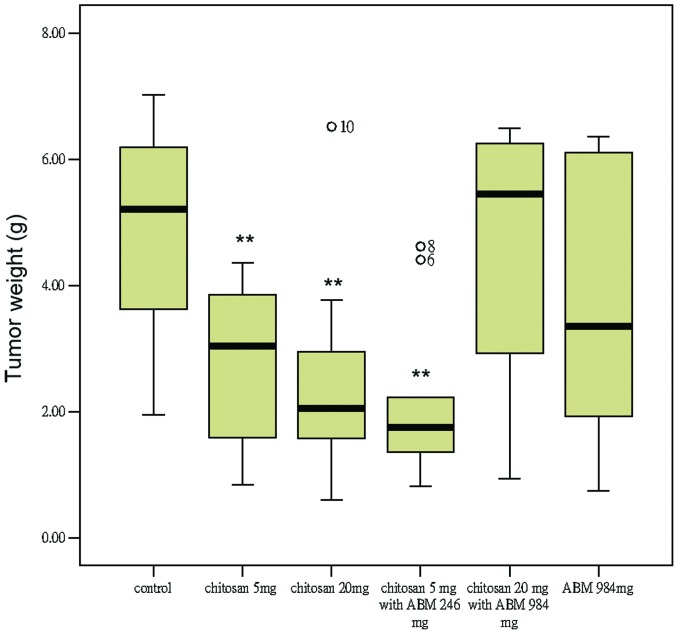
Groups 2, 3 and 4 reduced tumor growth compared with the control group. No significant differences were identified among these three effective groups. If sample 10 in group 3, and sample 6 and 8 in group 4 are excluded, the results demonstrated a greater reduction in tumor weight in groups 3 and 4 compared with group 2. Data are expressed as the mean ± standard deviation; ^*^P<0.05, ^**^P<0.01, ^***^P<0.001 vs. control. ABM, Agaricus blazei Murill.

In the present study, the results from the analysis of tumor weights suggested that the 5 mg chitosan (group 2), 20 mg chitosan (group 3) and 5 mg chitosan + 246 mg ABM (group 4) possessed anticancer activity. The survival rates were another important indicator. Following a 6-week treatment period, the survival rates of the rats in the three positive effective groups (3, 4 and 5) were all 100%. As no differences were observed in tumor weight among these groups, 5 mg chitosan was suggested as a first choice in treatment due to its low dose and single rather than combination therapy. However, if sample 10 in group 3 and samples 6 and 8 in group 4 were excluded, a greater reduction in tumor weight was observed in groups 3 and 4 compared with group 2. Therefore, 20 mg chitosan or 5 mg chitosan + 246 mg ABM were suggested as more effective doses for treatment compared with 5 mg (Fig. 3). This was further supported by the observation that groups 3 and 4, but not group 2, were able to reduce the levels of GOT (Table I).

Tumor weight is not proportional to tumor volume due to necrosis and cavitation of the inner tumor mass. In the present study, the tumor volumes were 2,849±1431, 1,764±877, 1,949±1581, 1,281±720, 2,352±1860 and 3,395±1934 mm^3^ in groups 1–6, respectively. Notably, the treatments in groups 2, 3 and 4 were able to reduce tumor volumes, of which group 4 was identified as the most effective. This suggested that 5 mg chitosan + 246 mg AMB was the optimal treatment strategy due to its reductions in tumor weight and volume.

Folkman *et al* (39) identified tumor angiogenesis as a potential target for the treatment of cancer, and studies have identified the VEGF-VEGFR system as the major regulator in tumor angiogenesis (40–42). Solid tumors often become hypoxic due to a rapid growth of tumor cells (43). Hypoxic stress is an important inducer of the *VEGF* gene via stabilization and activation of the hypoxia inducible factor (HIF) transcription factor; the 5′-upstream sequence of the *VEGF* gene has a HIF-response element motif, resulting in high levels of gene expression (44). A previous study by Kim *et al* (45) demonstrated that anti-human VEGF antibody efficiently suppressed the growth of human tumor xenografts transplanted into immune-deficient mice. This antibody can inhibit only the human-type VEGF, derived from tumor cells, and not the mouse VEGF, derived from the cells surrounding the tumor; however, tumor growth was significantly suppressed. These results suggested that tumor-derived VEGF is important in tumor angiogenesis. Although the majority of previous studies investigating VEGF and its receptors have focussed on their functions in angiogenesis and in endothelial cells, the function of VEGF in cancer biology appears to be an emerging area of importance (46). VEGF mediates vasculogenesis and angiogenesis through the promotion of endothelial cell growth, migration and mitosis, and is involved in the pathogenesis, progression and metastasis of cancer (47). The role of the VEGF signaling pathway in liver regeneration and tumor growth remains unclear, however, the use of antiangiogenic agents in combination with surgical treatment is almost certaily beneficial (48). In the present study, only treatment with 5 mg chitosan + 246 mg ABM was able to significantly reduce the levels of VEGF. Therefore, 5 mg chitosan + 246 mg ABM may be used as a first choice anticancer treatment, targeting VEGF-VEGFR signaling.

Our previous study reported that mice injected with Smmu 7721 cells in the dorsal area, followed by oral administration of ABM extract at low (22.5 mg), medium (90 mg) or high (900 mg) doses exhibited a dose-dependent effect on tumor growth (36). In the present study, the effects of treatment were not dose-dependent. ABM is able to absorb the heavy metals in soil or air. The products of ABM manufactured in Brazil are a higher quality compared with those of in Taiwan due to lead pollution. Chitosan is able to break down or excrete several types of pollutants (49–51). As ABM and chitosan are capable of inhibiting tumor growth, the aim of the present study was to investigate whether ABM extract was effective against tumor growth in mice, and to determine whether treatment with LMW chitosan combined with ABM was able to enhance the inhibition of hepatoma formation by SK-Hep 1 cells in SCID mice. To the best of our knowledge, this is the first study to demonstrate the inhibition of tumor growth by the combination of chitosan and ABM, and support further investigation on the anticancer effects of these natural compounds.

## Figures and Tables

**Table I:** Anticancer effects of low molecular weight chitosan oligosaccharides in combination with ABM extract on the reduction of hepatoma formation by SK-Hep 1 cells in SCID mice.

Group	Treatment (mg)	Tumor weight (g)	GOT (IU/l)	GPT (IU/l)	VEGF (ng/ml)
1	0	4.81±1.84	162±80	17±4	0.572±0.054
2	5 chitosan+0 ABM	2.83±1.23^a^ (P=0.0083)	148±69	35±31	0.510±0.136
3	20 chitosan+0 ABM	2.56±1.65^a^ (P=0.0065)	91±19^a^ (P=0.020)	15±4	0.520±0.140
4	5 chitosan+246 ABM	2.15±1.33^a^ (P=0.0015)	99±26^a^ (P=0.033)	14±4	0.459±0.096^a^ (P=0.0191)
5	20 chitosan+984 ABM	4.59±2.11	152±77	18±7	0.572±0.164
6	984 ABM	3.79±2.39	139±86	20±6	0.439±0.039^a^ (P=0.0167)

Values are expressed as the mean ± standard deviation.

a P<0.05, vs. group 1, determined using Student’s t-test. SCID, severe combined immunodeficiency; ABM, *Agaricus blazei* Murill; GOT, glutamic oxaloacetic transaminase; GPT, glutamic pyruvic transaminase; VEGF, vascular endothelial growth factor.

**Table II:** Presence of necrosis, hemorrhage and calcification determined by hematoxylin and eosin staining of 10 tumor samples from each treatment group.

Treatment	Pathology	1	2	3	4	5	6	7	8	9	10	Average
Control	Necrotic rate	0.6	0.4	0.3	0.3	0.25	0.6	0.6	0.4	0.3		0.42±0.15
	Hemorrhage	−	−	−	−	−	+	−	+	−		2/9
	Calcification	−	−	−	−	−	+	+	−	−		2/9
5 mg chitosan	Necrotic rate	0.2	0.2	0.3	0.3	0.2	0.3	0.3	0.3	0.4	0.3	0.3±0.1
	Hemorrhage	−	+	+	+	−	+	+	+	+	+	8/10
	Calcification	−	−	−	−	+	−	+	+	+	+	5/10
20 mg chitosan	Necrotic rate	0.2	0.3	0.2	0.3	0.2	0.3	0.3	0.3	0.2	0.2	0.3±0.1
	Hemorrhage	+	−	−	−	+	+	+	+	+	+	7/10
	Calcification	−	−	−	−	−	−	−	−	+	+	2/10
5 mg chitosan + 246 mg ABM	Necrotic rate	0.4	0.3	0.2	0.3	0.3	0.2	0.3	0.3	0.4		0.3±0.1
	Hemorrhage	+	+	−	+	−	+	−	+	+		6/9
	Calcification	−	−	−	−	+	−	+	+	−		3/9
20 mg chitosan + 984 mg ABM	Necrotic rate	0.2	0.2	0.3	0.3	0.4	0.2	0.3	0.2			0.3±0.1
	Hemorrhage	−	−	−	−	−	−	+	+			2/8
	Calcification	+	+	−	+	+	−	+	+			6/8
984 mg ABM	Necrotic rate	0.3	0.2	0.2	0.3	0.2	0.3	0.2				0.2±0.1
	Hemorrhage	−	−	+	−	+	+	+				4/7
	Calcification	−	−	+	−	−	+	+				3/7

Data are expressed as the necrotic rate, of which the average is the mean ± standard deviation), and presence (+) or absence (−) of hemorrhage or calcification. ABM, *Agaricus blazei* Murill.

## References

[1] Elsabee MZ, Abdou ES (2013). Chitosan based edible films and coatings. Mater Sci Eng C Mater Biol Appl.

[2] Rouget C (1859). Des substances amylacees dans le tissu des animaux. specialement les articutes (chitine). Comp Rend.

[3] Minke R, Blackwell J (1978). The structure of alpha-chitin. J Mol Biol.

[4] Kim S-K, Rajapakse N (2005). Enzymatic production and biological activities of chitosan oligosaccharides (COS): A review. Carbohydrate Polymers.

[5] Yin H, Du Y, Zhang J (2009). Low molecular weight and oligomeric chitosans and their bioactivities. Curr Top Med Chem.

[6] Donnelly LE, Barnes PJ (2004). Acidic mammalian chitinase-a potential target for asthma therapy. Trends Pharmacol Sci.

[7] Elias JA, Homer RJ, Hamid Q, Lee CG (2005). Chitinases and chitinase-like proteins in T(H)2 inflammation and asthma. J Allergy Clin Immunol.

[8] Kawada M, Hachiya Y, Arihiro A, Mizoguchi E (2007). Role of mammalian chitinases in inflammatory conditions. Keio J Med.

[9] Zhu Z, Zheng T, Homer RJ (2004). Acidic mammalian chitinase in asthmatic Th2 inflammation and IL-13 pathway activation. Science.

[10] Klokkevold PR, Vandemark L, Kenney EB, Bernard GW (1996). Osteogenesis enhanced by chitosan (poly-N-acetyl glucosaminoglycan) in vitro. J Periodontol.

[11] Ratanavaraporn J, Kanokpanont S, Tabata Y, Damrongsakkul S (2009). Growth and osteogenic differentiation of adipose-derived and bone marrow-derived stem cells on chitosan and chitooligosaccharide films. Carbohydrate Polymers.

[12] Koping-Hoggard M, Mel’nikova YS, Varum KM, Lindman B, Artursson P (2003). Relationship between the physical shape and the efficiency of oligomeric chitosan as a gene delivery system in vitro and in vivo. J Gene Med.

[13] Koping-Hoggard M, Varum KM, Issa M (2004). Improved chitosan-mediated gene delivery based on easily dissociated chitosan polyplexes of highly defined chitosan oligomers. Gene Ther.

[14] Oliveira EN, El Gueddari NE, Moerschbacher BM, Peter MG, Franco TT (2008). Growth of phytopathogenic fungi in the presence of partially acetylated chitooligosaccharides. Mycopathologia.

[15] Shahabuddin M, Toyoshima T, Aikawa M, Kaslow DC (1993). Transmission-blocking activity of a chitinase inhibitor and activation of malarial parasite chitinase by mosquito protease. Proc Natl Acad Sci U S A.

[16] Ribeiro MP, Espiga A, Silva D (2009). Development of a new chitosan hydrogel for wound dressing. Wound Repair Regen.

[17] Feng C, Sun G, Wang Z (2013). Transport mechanism of doxorubicin loaded chitosan based nanogels across intestinal epithelium. Eur J Pharm Biopha.

[18] Yeh MY, Wu MF, Shang HS (2013). Effects of chitosan on xenograft models of melanoma in C57BL/6 mice and hepatoma formation in SCID mice. Anticancer Res.

[19] Kim HM, Hong SH, Yoo SJ, Baek KS, Jeon YJ, Choung SY (2006). Differential effects of chitooligosaccharides on serum cytokine levels in aged subjects. J Med Food.

[20] Karagozlu MZ, Kim SK (2014). Anticancer effects of chitin and chitosan derivatives. Adv Food Nutr Res.

[21] Lindequist U, Niedermeyer TH, Jülich WD (2005). The pharmacological potential of mushrooms. Evid Based Complement Alternat Med.

[22] Hawksworth DL (2001). Mushrooms: the extent of the unexplored potential. Int J Med Mushrooms.

[23] Hearst R, Nelson D, McCollum G (2009). An examination of antibacterial and antifungal properties of constituents of Shiitake (Lentinula edodes) and oyster (Pleurotus ostreatus) mushrooms. Complement Ther Clin Pract.

[24] Lindequist U, Teuscher E, Narbe G (1990). Neue Wirkstoffe aus Basidiomyceten. Z Phytother.

[25] Firenzuoli F, Gori L, Lombardo G (2008). The Medicinal Mushroom Agaricus blazei Murrill: Review of Literature and Pharmaco-Toxicological Problems. Evid Based Complement Alternat Med.

[26] Kahlos K, Kangas L, Hiltunen R (1987). Antitumor activity of some compounds and fractions from an n-hexane extract of Inonotus obliquus in vitro. Acta Pharm Fennica.

[27] Burczyk J, Gawron A, Slotwinska M, Smietana B, Terminska K (1996). Antimitotic activity of aqueous extracts of Inonotus obliquus. Boll Chim Farm.

[28] Chihara G, Maeda Y, Hamuro J, Sasaki T, Fukuoka F (1969). Inhibition of mouse sarcoma 180 by polysaccharides from Lentinus edodes (Berk.). sing Nature.

[29] Mizuno T (1999). The extraction and development of antitumor-active polysaccharides from medicinal mushrooms in Japan (review). Int J Med Mushrooms.

[30] Wasser SP, Weis AL (1999). Medicinal properties of substances occurring in higher Basidiomycetes mushrooms: current perspectives (review). Int J Med Mushrooms.

[31] Reshetnikov SV, Wasser SP, Tan KK (2001). Higher basidiomycetes as a source of antitumor and immunostimulating polysaccharides (review). Int J Med Mushrooms.

[32] Fujimiya Y, Suzuki Y, Oshiman K (1998). Selective tumoricidal effect of soluble proteoglucan extracted from the basidiomycete, Agaricus blazei Murill, mediated via natural killer cell activation and apoptosis. Cancer Immunol Immunother.

[33] Ito H, Shimura K, Itoh H, Kawade M (1997). Antitumor effects of a new polysaccharide-protein complex (ATOM) prepared from Agaricus blazei (Iwade strain 101) ‘Himematsutake’ and its mechanisms in tumor-bearing mice. Anticancer Res.

[34] Patel S, Goyal A (2012). Recent developments in mushrooms as anti cancer therapeutics: a review. 3 Biotech.

[35] Wu MF, Chen YL, Lee MH (2011). Effect of Agaricus blazei Murrill extract on HT 29 human colon cancer cells in SCID mice in vivo. In Vivo.

[36] Wu MF, Lu HF, Hsu YM (2011). Possible reduction of hepatoma formation by Smmu 7721 cells in SCID mice and metastasis formation by B16F10 melanoma cells in C57BL/6 mice by Agaricus blazei murill extract. In Vivo.

[37] Chen WT, Yang CL, Yin MC (2014). Protective effects from Houttuynia cordata aqueous extract against acetaminophen-induced liver injury. Biomedicine.

[38] Jin CY, Moon DO, Choi YH, Lee JD, Kim GY (2007). Bcl-2 and caspase-3 are major regulators in Agaricus blazei-induced human leukemia U937 cell apoptosis through dephoshorylation of Akt. Biol Pharm Bull.

[39] Hanahan D, Folkman J (1996). Patterns and emerging mechanisms of the angiogenic switch during tumorigenesis. Cell.

[40] Shibuya M (2011). Involvement of Flt 1 (VEGF receptor-1) in cancer and preeclampsia. Proc Jpn Acad Ser B Phys Biol Sci.

[41] Shibuya M, Claesson-Welsh L (2006). Signal transduction by VEGF receptors in regulation of angiogenesis and lymphangiogenesis. Exp Cell Res.

[42] Alitalo K, Carmeliet P (2002). Molecular mechanisms of lymphangiogenesis in health and disease. Cancer Cell.

[43] Li Y, Fu L, Li JB (2014). Increased expression of EIF5A2, via hypoxia or gene amplification, contributes to metastasis and angiogenesis of esophageal squamous cell carcinoma. Gastroenterology.

[44] Shibuya M (2013). Vascular endothelial growth factor and its receptor system: physiological functions in angiogenesis and pathological roles in various diseases. J Biochem.

[45] Kim KJ, Li B, Winer J (1993). Inhibition of vascular endothelial growth factor-induced angiogenesis suppresses tumour growth in vivo. Nature.

[46] Perrot-Applanat M, Di Benedetto M (2012). Autocrine functions of VEGF in breast tumor cells: adhesion, survival, migration and invasion. Cell Adh Migr.

[47] Wang K, Peng HL, Li LK (2012). Prognostic value of vascular endothelial growth factor expression in patients with prostate cancer: a systematic review with meta-analysis. Asian Pac J Cancer Prev.

[48] Eveno C, Pocard M (2012). VEGF levels and the angiogenic potential of the microenvironment can affect surgical strategy for colorectal liver metastasis. Cell Adh Migr.

[49] Li M, Xu J, Li R (2014). Simple preparation of aminothiourea-modified chitosan as corrosion inhibitor and heavy metal ion adsorbent. J Colloid Interface Sci.

[50] Liu J, Wen XY, Lu JF, Kan J, Jin CH (2014). Free radical mediated grafting of chitosan with caffeic and ferulic acids: Structures and antioxidant activity. Int J Biol Macromol.

[51] Seo DJ, Nguyen DM, Park RD, Jung WJ (2014). chitosan-cinnamon beads enhance suppressive activity against Rhizoctonia solani and Meloidogyne incognita in vitro. Microb Pathog.

